# Optimum blue light exposure: a means to increase cell-specific productivity in Chinese hamster ovary cells

**DOI:** 10.1007/s00253-024-13363-4

**Published:** 2024-12-05

**Authors:** Stefanie Föller, Niklas Regett, Levin Lataster, Gerald Radziwill, Ralf Takors

**Affiliations:** 1 Institute of Biochemical Engineering, University of Stuttgart, Allmandring 31, 70569 Stuttgart, Germany; 2 Institute of Biology II, University of Freiburg, 79098 Freiburg, Germany

**Keywords:** CHO cells, Cell culture, Recombinant proteins, Antibodies, Cell-specific productivity

## Abstract

**Abstract:**

Research for biopharmaceutical production processes with mammalian cells steadily aims to enhance the cell-specific productivity as a means for optimizing total productivities of bioreactors. Whereas current technologies such as pH, temperature, and osmolality shift require modifications of the cultivation medium, the use of optogenetic switches in recombinant producer cells might be a promising contact-free alternative. However, the proper application of optogenetically engineered cells requires a detailed understanding of basic cellular responses of cells that do not yet contain the optogenetic switches. The knowhow of ideal light exposure to enable the optimum use of related approaches is missing so far. Consequently, the current study set out to find optimum conditions for IgG1 producing Chinese hamster ovary (CHO) cells which were exposed to blue LED light. Growth characteristics, cell-specific productivity using enzyme-linked immunosorbent assay, as well as cell cycle distribution using flow cytometry were analyzed. Whereas too harsh light exposure causes detrimental growth effects that could be compensated with antioxidants, a surprising boost of cell-specific productivity by 57% occurred at optimum high light doses. The increase coincided with an increased number of cells in the G1 phase of the cell cycle after 72 h of illumination. The results present a promising new approach to boost biopharmaceutical productivity of mammalian cells simply by proper light exposure without any further optogenetic engineering.

**Key points:**

*• Blue LED light hinders growth in CHO DP-12 cells*

*• Antioxidants protect to a certain degree from blue light effects*

*• Illumination with blue LED light raises cell-specific productivity*

**Supplementary Information:**

The online version contains supplementary material available at 10.1007/s00253-024-13363-4.

## Introduction

Biopharmaceuticals play an important role in today’s medical treatments and healthcare. The market share of biopharmaceuticals, mainly monoclonal antibodies, increased vastly over the last decade, and is also forecasted to increase further in the future (Walsh and Walsh 2022; Farid et al. 2020; Lu et al. 2020), although mRNA technologies (Urquhart 2022) as well as bispecific antibodies (Ma et al. 2021; Lu et al. 2020) are gaining momentum. Coinciding with the rising market volumes, productivities should equally rise to meet the demand. In the past, this was achieved predominately by improving volumetric productivities, i.e., improvements of cultivation conditions for increasing viable cell density and consequently volumetric product formation rates. Nowadays, antibody titers of 5 g/L up to well over 10 g/L can be reached (Liang et al. 2023; Mahé et al. 2022; Handlogten et al. 2018; Shukla et al. 2017), especially using perfusion processes (McDonnell et al. 2022; Liang et al. 2023; Ding et al. 2022). However, such approaches may reach technical limits of bioreactors (Ozturk 1996) leading to an increased focus on cell-specific productivity (Farid et al. 2020; Ozturk 1996; Becker et al. 2019a, b; Wijaya et al. 2021; Verhagen et al. 2020a, b; Torres and Dickson 2022). Previous research indicated a correlation between cell-specific productivity and the cell cycle. Mainly, the G1 phase was associated with an increase of cell-specific productivity (Hendrick et al. 2001; Dutton et al. 2006; Park et al. 2016; Fussenegger et al. 1997). Chemicals such as dimethyl sulfoxide (Fiore and Degrassi 1999; Fiore et al. 2002), sodium butyrate (Paterson et al. 1994; Oh et al. 1993; Gorman et al. 1983; Herz and Halwer 1982; Qiu et al. 2017; D'Anna et al. 1980), and valproic acid (Li et al. 2005; Catalano et al. 2005; Backliwal et al. 2008) have been investigated to arrest cells in G1 and lead to an improved productivity.

Other methods to increase cell-specific productivity in bioprocesses include parameter shifts of pH (Becker et al. 2019b; Ivarsson et al. 2015; Yoon et al. 2005), temperature (Ahn et al. 2008; Kaufmann et al. 1999; Martínez et al. 2015; Yoon et al. 2003), and osmolality (Kiehl et al. 2011; Herz and Halwer 1982; Oh et al. 1993). On the downside, all those methods also trigger various stress responses inside the cell. This can result in altered glycosylation patterns of the product, diminished cell growth and viability due to apoptosis, and therefore, a lower overall product yield in the reactor.

Another alternative is the putative modulation of cell cycle by the use of optogenetics. The implementation of optogenetic switches opens the door for modulating cellular regulation finally yielding increased productivity in bioprocesses. First approaches have been made in microbial bioprocesses (Hoffman et al. 2021; Hörner et al. 2019; Komera et al. 2022; Toya and Shimizu 2020; Wang et al. 2018; Zhao et al. 2018; Bacchus and Fussenegger 2012), and potential applications in mammalian processes are being discussed (Pouzet et al. 2020; Minami and Shah 2021; Mansouri et al. 2019; Bacchus and Fussenegger 2012). Optogenetic switches make use of light responsive proteins, which undergo a conformational change when illuminated by their corresponding wavelength. Whereas optogenetic switches for multiple wavelengths, including UV exist, mostly blue light switches are used (see optobase.org (Kolar et al. 2018)).

Exposing cells to light requires the careful assessment of multiple factors affecting cellular performance and stability. The energy impact of blue light, with a wavelength between 450 and 495 nm, is ranked at the top of the visible light spectrum only exceeded by non-visible UV light. The radiation exposure has effects on all kinds of molecules and tissue (Sheraz et al. 2014; Nixon and Wang 1977; Nakashima et al. 2017). Already in the 1970s, research has been conducted to investigate the effect fluorescent lab light or bilirubin light can have on tissue cultures or on medium components (Sheraz et al. 2014; Schnellbaecher et al. 2019; Edwards et al. 1994; Nixon and Wang 1977; Wang 1975, 1976; Zigler et al. 1985). Also, recent publications document the effect of blue light on cells, outlining reduced growth and the occurrence of intracellular reactive oxygen species (ROS), depending on the applied light dose (Wall et al. 2019; Walsh et al. 2021). While ROS has essential functions inside the cells such as signal transduction (Reczek and Chandel 2015; Finkel 2011), it may cause many detrimental effects if present in abundance (Milkovic et al. 2019). Intrinsically, mammalian cells make use of antioxidants to counter ROS. The most abundant antioxidant inside a cell is reduced glutathione (GSH), which reduces superoxides. Oxidized glutathione (GSSG) is then either reduced via glutathione reductase to replenish GSH pools, or shuttled outside the cell (Georgiou-Siafis and Tsiftsoglou 2023; Lushchak 2012).

In addition to the energy impact related to the chosen wavelength, the duration of cellular radiation exposure should be considered, too. Typically, laboratory settings for investigating optogenetic tools vary significantly from those of potential bioprocess applications. Whereas mammalian cells are often investigated as a monolayer in micro-titer plates (or alike), being exposed to light stimuli only for short time intervals (e.g., pulses of a few seconds over a timeframe of 24 h (Antwi et al. 2023)), the same mammalian constructs should also be robust enough to perform optimally for 7–15 days in high-cell density bioreactor cultivations. Furthermore, lab tests may still contain up to 10% fetal bovine serum whereas mammalian bioprocesses solely apply chemically defined medium (Ritacco et al. 2018; Fletcher and Harris 2016) comprising high amounts of riboflavin, which is particularly susceptible to blue light (Sheraz et al. 2014; Schnellbaecher et al. 2019).

Therefore, experiments are necessary to unravel the fundamental impact of typically applied light stimuli under realistic cell culture conditions. Such studies provide the groundwork to engineer optogenetically modulated cells for bioproduction purposes. To this end, the Chinese hamster ovary (CHO) cell line CHO DP-12, which produces and secretes an antibody against interleukin-8 (IL-8), has been chosen as a showcase. The current study set out to investigate optimal lighting conditions for the implementation of optogenetic switches in a bioreactor setting. Surprisingly, the non-optogentically manipulated cells demonstrated phenotypes of maximized csp under optimal light exposure. All cultivation investigations employ a chemically defined medium and account for the influence of blue light as a promising stimulus for optogenetic installations.

## Material and methods

### Mini bioreactor cultivation of cells

The CHO DP-12 cells (kindly provided by Prof. Thomas Noll; ATCC® CRL 12445™) were cultured in a shaking incubator at 150 rpm with 50-mm displacement (minitron, infors HT) at 37 °C, 5% CO_2_ in a humid atmosphere and at an angle of around 50° for better aeration. Chemically defined TC-42 medium (Xell) in a 50-mL mini bioreactor (Corning) with a filter cap was used. Cells were seeded at 0.2–0.3 × 10^6^ cells/mL. For the experiment with the antioxidants, a seeding density of ~ 0.1 × 10^6^ cells/mL was chosen to allow for a longer exponential growth timeframe.

### Determination of cell density and viability

For the determination of cell concentration and viability, a device for holographic measurement was used (fluidlab R-300, anvajo). Twenty microliter of cell suspension was pipetted into an acella 100 microscopic slide (anvajo) and analyzed.

### Illumination of cells

Waterproof RGB LED strips (VARDAflexIP68, rutec) were wrapped around the test tube rack inside the shaking incubator, and connected to a Raspberry Pi 4 (raspberry pi). The LED strips were controlled via a Python script (Python Software Foundation, https://www.python.org/). Examined lighting conditions were intensity of 3 W/m^2^, 6 W/m^2^, or 12 W/m^2^ and an interval of 1-min illumination, 29-min dark phase (1/29); 5-min illumination, 25-min dark phase (5/25); and 10-min illumination, 20-min dark phase (10/20). Intensities are averages, as the light installation for suspension cultures does not allow for equal light distance for all sides of the minibioreactors.

### Intracellular ROS measurement

For the intracellular ROS measurement, CellROX Green reagent (Life Technologies Corporation) was used. 1 × 10^6^ live cells per well were harvested, centrifuged, and resuspended in 150 μL PBS. 0.3-μL CellROX Green reagent was added and incubated for 30 min at 37 °C. Cells were washed 3 times with PBS and finally resuspended in 150-μL PBS and measured at Ex:508 nm/Em:525 nm.

### Cell cycle analysis

1 × 10^6^ cells were washed in ice-cold PBS, fixed with ice-cold fixation buffer (70% ethanol, 30% PBS) and stored at − 20 °C until investigation. Cells were washed twice with PBS + 1% albumin, and the cell pellet was resuspended in staining buffer (propidium iodide (PI) and RNase in PBS). After 10-min incubation in the dark, the cells were examined with a MACSQuant Analyzer with a 610/20-nm filter and 50,000 events. Data were analyzed with FACSalyzer software.

### Enzyme-linked immunosorbent assay against IgG-antibody

The concentration of secreted antibody was determined with an enzyme-linked immunosorbent assay (ELISA). High-binding 96-well plates were coated with an anti-human IgG F(c) (goat) antibody (Rockland Immunochemicals). Free binding sites were blocked with 1% BSA. Diluted supernatants and standards were pipetted into the coated wells in duplicates. Detection was done with a horseradish peroxidase coupled anti-human kappa chain (goat) antibody (Rockland Immunochemicals) and TMB substrate (SeramunBlau, Seramun Diagnostica). After stopping the reaction, the absorption was measured at 450 nm and 620 nm for background (Tecan Spark, Tecan Trading, Ltd.).

### Statistical methods

Error bars show standard deviations that were calculated based on three biological replicates. Unpaired one-sided Student's t test was performed to investigate the data for statistical significance (***p = 0.001, **p = 0.01, *p = 0.05).

## Results

### Reduced growth of cells caused by blue light can be prevented by addition of reduced l-glutathione

**Fig. 1:**
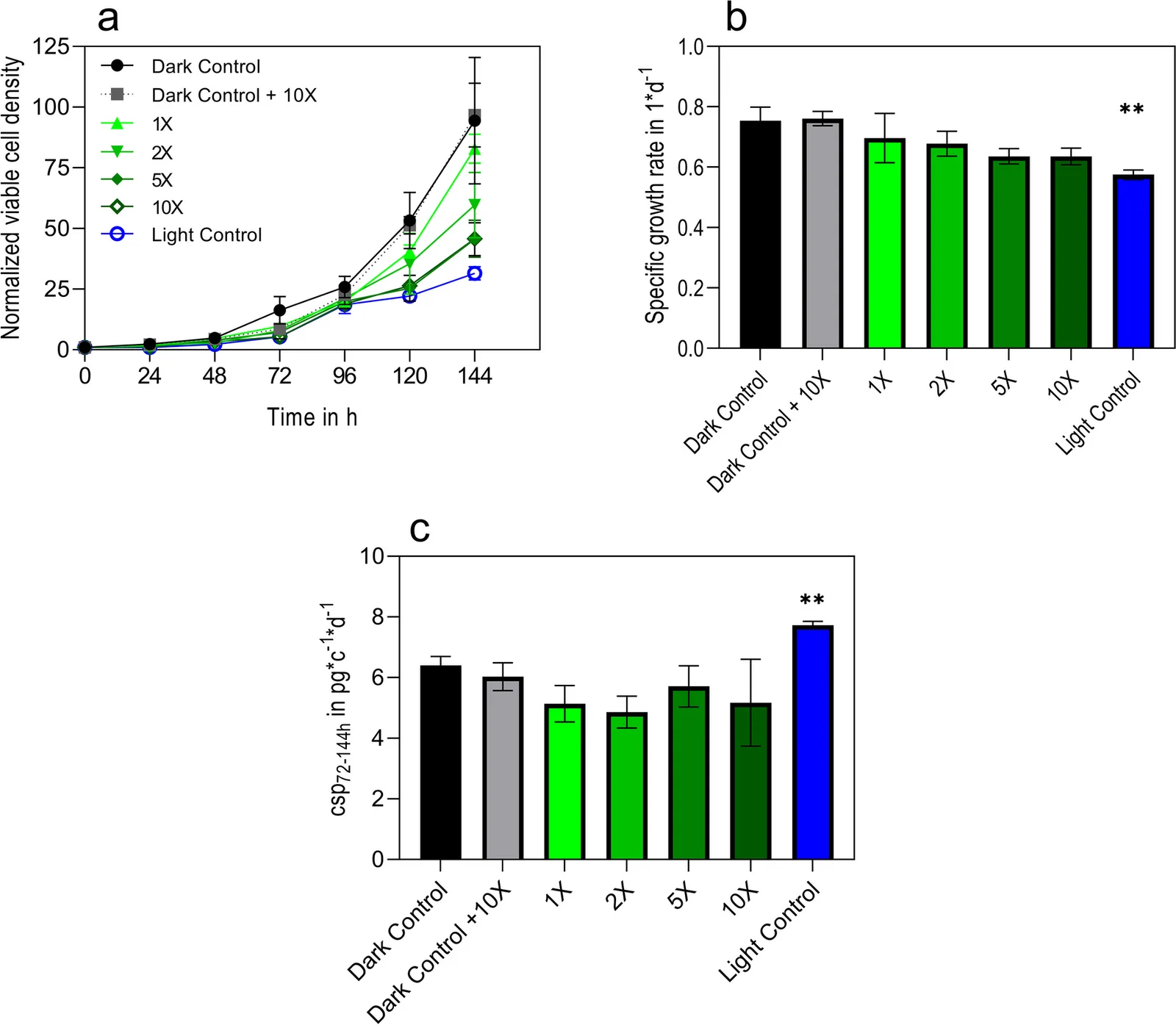
CHO DP-12 illuminated by blue LED light and treated with reduced glutathione. Viable cell density measured via holographic microscopy, normalized to the starting point, representing x-fold changes (**a**). Calculated specific growth rate over the course of the whole cultivation in 1*d^−1^ (**b**). Cell-specific productivity (csp) calculated via the antibody titer detected by enzyme-linked immunosorbent assay and subsequently divided by the integral viable cell concentration in the timeframe of 72–144 h and normalized to the control (**c**). Light green to dark green represent the different reduced l-glutathione concentrations of 65 μM (1 × , ▲), 130 μM (2 × , ▼), 325 μM (5 × , ♦), and 650 μM (10 × , ◯), respectively. Cells were illuminated for an interval of 1 min blue LED light and 29-min dark phase, repeated over 144 h and an intensity of 3 W/m.^2^. Control cells that were illuminated with no added antioxidant are shown in blue (◯). Control cells that were kept in the dark were untreated (black, ●) or treated with the maximum concentration of 650-μM reduced l-glutathione (grey, ■). Error bars show standard deviations of biological triplicates. Significance was tested with one-sided *t* test. **p = 0.01

Since blue light was reported to increase intracellular ROS formation, antioxidants may mitigate the detrimental effects on growth. For this experiment, reduced l-glutathione, a well-known antioxidant, was used. CHO DP-12 cells were illuminated with blue LED light in intervals of 1-min illumination, followed by 29-min darkness, over the course of 144 h, at an illumination intensity of 3 W/m^2^. For a starting concentration of reduced glutathione, 65 μM (1 ×) was used (Parshad et al. 1978). To scan for the most ideal concentration, this value was increased double (2 ×), fivefold (5 ×), and tenfold (10 ×). The viable cell density was measured via holographic measurement, the corresponding specific growth rate was calculated, and the cell-specific productivity was determined using an enzyme-linked immunosorbent assay (ELISA) (Fig. 1).

Whereas cells in the light control reach a 30-fold increase in viable cell density within 144 h, the cells treated with 65-μM reduced l-glutathione achieved a cell density comparable to the cells kept in the dark with an 82.6-fold and a 96-fold increase in viable cell density, respectively (Fig. 1a). This growth phenotype is also reflected by the specific growth rates. The light control reaches 0.57 1/days whereas the cells in the dark controls and the cells treated with 65-μM reduced l-glutathione grew faster than 0.7 1/days (Fig. 1b). When examining the cell-specific productivity, the cells illuminated by blue LED light in the absence of reduced glutathione produced antibodies at ~ 28% and 50% greater rate than the cells of the dark control and the reduced l-glutathione treated, illuminated cells, respectively (Fig. 1c).

### Blue LED light slows down growth of CHO DP-12 cells depending on intensity and lighting interval

**Fig. 2:**
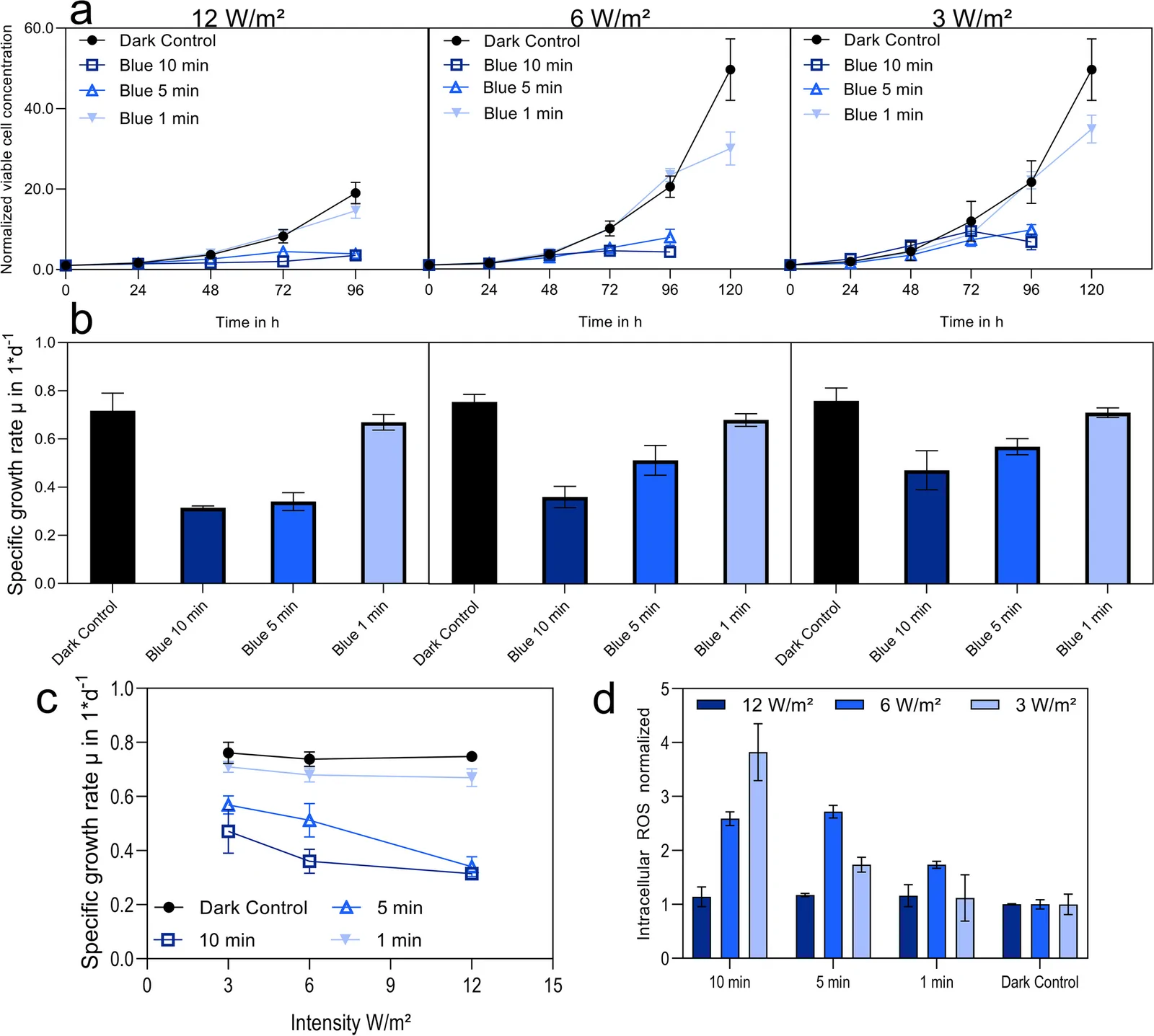
CHO DP-12 illuminated by blue LED light with different illumination intensities and intervals. Viable cell density measured via holographic microscopy, normalized to the starting point, equaling x-fold changes (**a**). Calculated specific growth rate over the course of the whole cultivation in 1*d^−1^ (**b**). Specific growth rate in 1*d^−1^ over the light intensity in W/m^2^ (**c**). Intracellular ROS measured via CellROX green after 96-h cultivation time (**d**). Longest lighting interval 10-min illumination/20-min dark phase (dark blue, ☐), medium lighting interval 5-min illumination/25-min dark phase (medium blue, △), short lighting interval 1-min illumination/29-min dark phase (light blue, ▼), and control cells kept in the dark (black, ●). Error bars show standard deviations of biological triplicates

To investigate the enhancement effect of blue light on cell-specific productivity and also the detrimental effect on growth of CHO cells, different illumination interval and intensity combinations were tested (Table 1). Because higher cell density suspension cultures require a higher light intensity to penetrate the cell suspension, a maximum of 12 W/m^2^, a medium intensity of 6 W/m^2^, and a low intensity of 3 W/m^2^ were tested. In combination, three different lighting intervals of 10-min illumination, 20-min dark phase; 5-min illumination, 25-min dark phase; and 1-min illumination, 29-min dark phase over the course of the cultivation, were examined. The above-mentioned combinations of intensity and lighting intervals result in different total light doses. Samples were taken every 24 h for 96 h. For the medium and low intensity, combined with the shortest interval, an additional 24 h was investigated until changes in growth, compared to the dark control, were seen. To determine the amount of intracellular ROS, a CellROX assay was performed for the cells at the end of the cultivation at 96 h after being illuminated at an intensity of 12 W/m^2^, 6 W/m^2^, or 3 W/m^2^ at different time intervals of 10 min, 5 min, or 1 min every half an hour (Fig. 2).

**Table 1:** Light settings used and the resulting total light dose

Light intensity (W/m^2^)	Illumination time (h)	Total light dose (W*h/m^2^)
12	0.33	4.0
	0.166	2.0
	0.033	0.4
6	0.33	2.0
	0.166	1.0
	0.033	0.2
3	0.33	1.0
	0.166	0.5
	0.033	0.1

Scanning different light intensities and intervals revealed the effect of blue light on CHO DP-12 cells (Fig. 2). All light settings resulted in a diminishing growth curve for the CHO DP-12 cells. Cells illuminated for intervals of 1 min behaved similarly to cells that were kept in the dark, showing a 20-fold increase in viable cell density within 96 h. Cells illuminated for intervals of 5 or 10 min, irrespective of the intensity of the blue light, only exhibited a 3.5-fold to 9.7-fold rise in viable cell density. The highest intensity of 12 W/m^2^ reduced the growth to around 50% for both the 10-min interval as well as the 5-min interval. Resulting in a decrease in the specific growth rate from 0.75 to 0.32 1/day and 0.36 1/day, respectively. A correlation between the lighting intervals and the specific growth rate could be observed for 12 W/m^2^ and 6 W/m^2^ (Fig. 2c). Intracellular ROS increased proportionally with illumination interval for the lowest intensity. This effect decreased with higher intensities. However, for the highest light intensity of 12 W/m^2^, no changes in intracellular ROS could be observed when compared to the control (Fig. 2d).

### Blue LED light increases cell-specific productivity through illumination

**Fig. 3:**
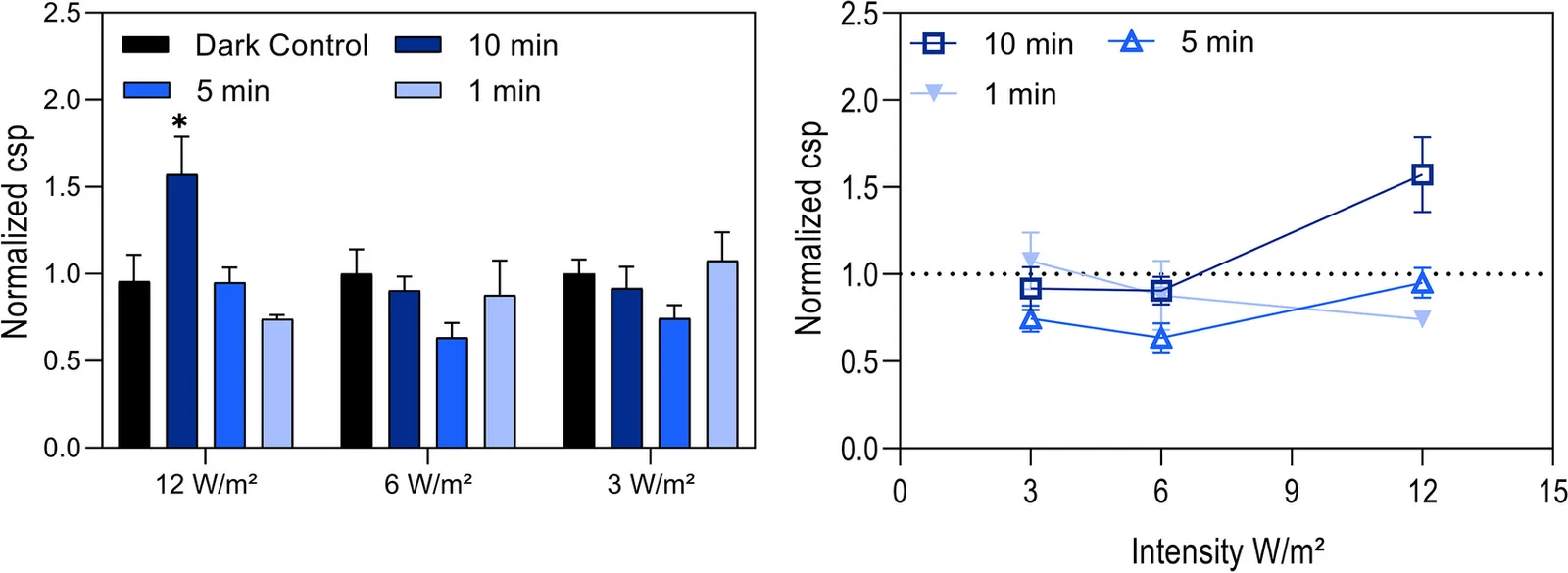
Cell-specific productivity (csp) between 48 and 96 h. Cell-specific productivity (csp) calculated via the antibody titer detected by enzyme-linked immunosorbent assay and subsequently divided by the integral viable cell concentration in the timeframe of 48–96 h and normalized to the control. Longest lighting interval 10-min illumination/20-min dark phase (dark blue, ☐), medium lighting interval 5-min illumination/25-min dark phase (medium blue, △), short lighting interval 1-min illumination/29-min dark phase (light blue, ▼), and control cells kept in the dark (dotted black line). Error bars show standard deviations of biological triplicates. Significance was tested with one-sided *t* test. *p = 0.05

Next, the cell-specific productivity of the CHO DP-12 cells was calculated, determining the secreted antibody using an enzyme-linked immunosorbent assay (ELISA) for the different lighting settings (Fig. 3).

Cell-specific productivity increased with the highest lighting intensity and the longest lighting interval. The highest total light dose raised the cell-specific productivity of the cells by 57% compared to the control cells. The other light doses did not induce a positive effect on cell-specific productivity in the cells.

### An increased number of cells arrest in the G1 phase of the cell cycle when illuminated by blue light

**Fig. 4:**
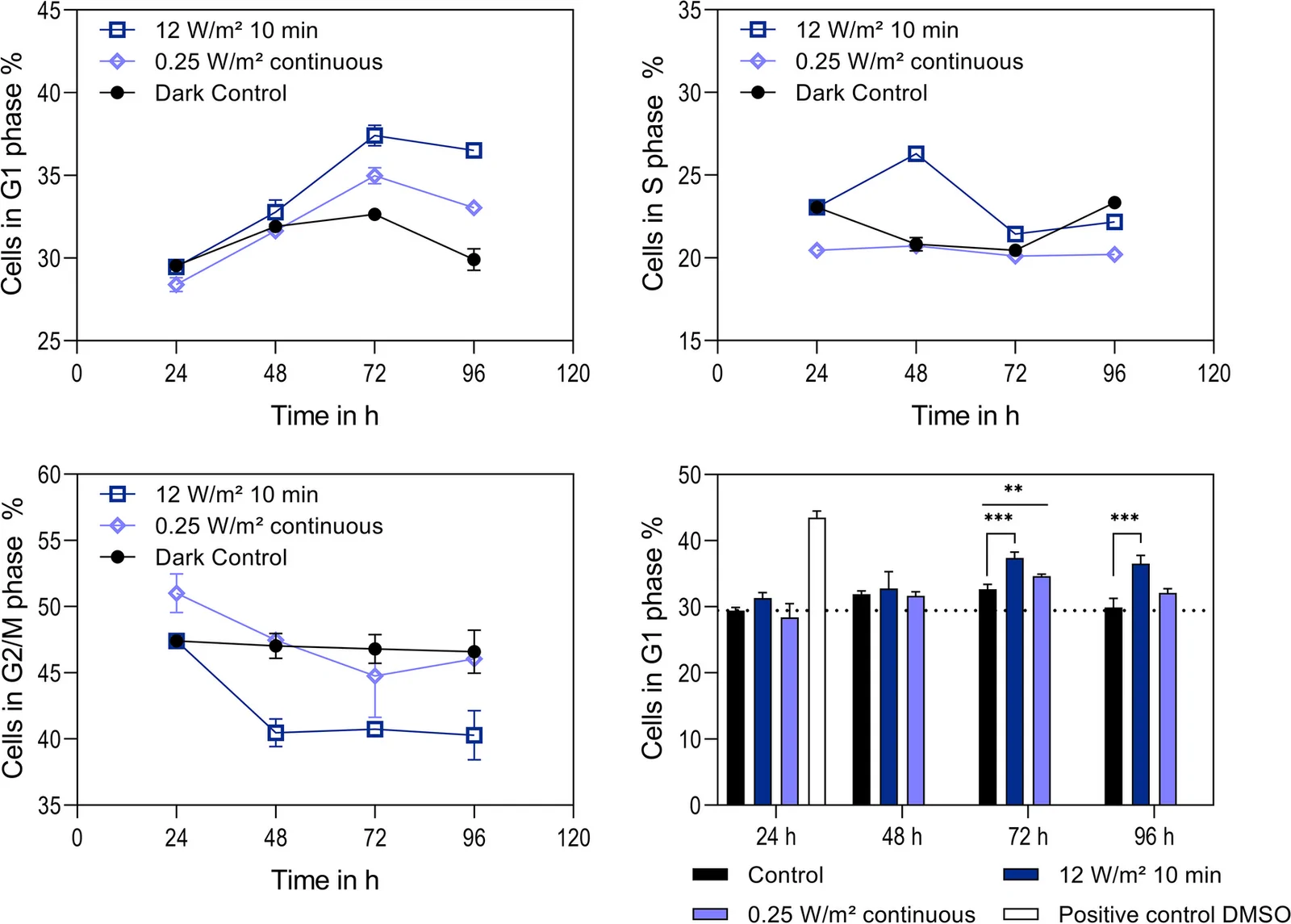
Cell cycle phase distribution of CHO DP-12 cells. Percentage of cells in G1 phase, S phase, and G2/M phase for cells kept in the dark (dark control, black, ●), cells illuminated with 12 W/m^2^ for 10-min intervals (12 W/m^2^ 10 min, dark blue, ☐), and cells illuminated permanently with 0.25 W/m^2^ (0.25 W/m.^2^ permanent, light blue, ◇). (B) G1 phase distribution compared to a positive control cells treated with 2% dimethyl sulfoxide (DMSO) for 24 h. Dashed line shows baseline value of cells in G1 phase at 0 h. Error bars show standard deviations of biological triplicates. Significance was tested with one-sided *t* test. **p = 0.01, ***p = 0.001

As increased antibody production could be observed, experiments were conducted to search for the possible reason. As Wall et al. have shown a correlation between illumination of cells by blue light and increased intracellular ROS as well as arrest of the cells in G1 phase of the cell cycle (Wall et al. 2019), the cell cycle was examined with different light settings. As the highest intensity of 12 W/m^2^ and the longest interval of 10 min have shown the highest increase of cell-specific productivity, the cells were examined for cell cycle arrest (Fig. 4).

An increased fraction of cells being in G1 phase was observed after illumination. Whereas all conditions show an increase during the course of the cultivation, displaying a peak after 72 h, the cells illuminated at a light dose of 4 W*h/m^2^ exhibited the greatest fraction of cells in G1 phase. At 72 h, 37.4% of cells were in G1 phase. A permanent illumination at a low intensity resulted in 34.9% of the cells in G1 phase, presenting an increase of 8% and 5.5% to the baseline at 0 h, respectively. The fraction of cells in S phase varied only slightly in all approaches. The fraction of cells in G2/M phase varied inversely to the G1 phase for illuminated cells.

## Discussion

### Reduced l-glutathione prevents detrimental effect of light in CHO suspension cultures

Increased intracellular ROS occurs after illumination of cells (Wall et al. 2019; Walsh et al. 2021). The antioxidant ascorbic acid 2-glucoside, an ascorbic acid stabilized with glucose, has been demonstrated to protect cells from light induced cytotoxicity (Walsh et al. 2021). Another likely candidate is reduced l-glutathione. Glutathione is a well-known cellular and abundant antioxidant. Supplementation studies adding reduced l-glutathione and l-ascorbic acid have been conducted (data not shown) following related previous tests (Parshad et al. 1978). However, neither the joint nor the sole application of l-ascorbic acid improved cell growth in the light-exposure settings.

Interesting enough, although the applied reduced l-glutathione concentration of 65 μM was motivated by tissue culture studies of the 1970s (Parshad et al. 1978), the concentration also revealed growth benefits for CHO DP-12 cells in suspension cultures being illuminated with blue LED light (Fig. 1). Increasing the concentration further, however, did not yield any additional positive effects. In fact, higher concentrations of 325 μM and 650 μM led to a decline in cell growth, nearly matching the growth levels of the illuminated control cells. Notably, in the absence of light, high glutathione levels did not adversely affect growth. This observation suggests that the oxidized form of glutathione, potentially generated by blue light exposure, may inhibit growth (Nakashima et al. 2017).

The preferred range for cytosolic glutathione levels is 1–10 mM (Meister 1988), although even micromolar concentrations have been reported (Cantin et al. 1987; Sutherland et al. 1985). This indicates that cells can tolerate elevated l-glutathione levels, provided that blue light exposure does not impose excessive stress. Notably, the protective effects of reduced l-glutathione were only observed under an intensity of 3 W/m^2^ with 1-min intervals. At higher intensities and longer intervals, the antioxidant’s protective properties diminished, likely due to l-glutathione oxidation in the presence of blue light (Nakashima et al. 2017) or because the increased light intensity induced excessive stress within the cells. The DNA damage and endoreduplication previously reported (Wall et al. 2019; Walsh et al. 2021) are likely not solely caused by intracellular ROS levels.

In summary, reduced l-glutathione is a promising protective agent when cells are exposed to blue light. However, proper levels must be installed in a dose-dependent manner.

### Blue LED light affects the growth of CHO suspension cultures

All lighting regimes induced slowed down growth depending on the total light dose (product of lighting intensity and interval) (Table 1; Fig. 2). Although the lowest light dose of 0.1 and 0.2 W*h/m^2^ only created an effect after 120 h, effects of other light doses were observed earlier, i.e., within 96 h. Interestingly, even though cell growth almost stopped after 96 h exposure to 4 W*h/m^2^, viability did not drop significantly below 90% (data not shown), the threshold value for a culture being physiologically in good shape. Although viability was not affected considerably here, the highest light dose has an impact on cell-specific growth rate, mirroring effects of either hypothermia (Becerra et al. 2012) or treatment with high doses of methylthioadenosine (MTA) (Verhagen et al. 2020b).

### Effect of blue LED light on cell-specific productivity is dependent on total light dose

The experiments clearly outline a positive stimulation on cell-specific productivity when the cells are illuminated with a suitable light dose (Figs. 1 and 3). While a high light dose has demonstrated a greater effect within 96 h (Fig. 3), another experiment over a longer period of 144 h affected the cell-specific productivity with lower light doses (Fig. 1). Although a light dose of 4 W*h/m^2^ increased the cell-specific productivity by around 57%, a decrease in cell growth of around 50% was observed when compared to the control (Fig. 2b). At a light dose of 0.1 W*h/m^2^, cell growth was affected after 120 h, resulting in a reduced specific growth rate of 24% after 144 h cultivation time (Fig. 1a, b). The cell-specific productivity between 72 and 144 h increased by 18% in the cells that are illuminated (Fig. 1c).

Even though the settings examined increase the cell-specific productivity, the overall volumetric yield suffers due to the low cell density present in the cultivation.

Given that cell viabilities remained high even at the highest light dose, it stands to reason that the cell cycle might have been arrested because of illumination (Walsh et al. 2021; Wall et al. 2019). Indeed, the results demonstrate that cells accumulated in G1 phase over a 96-h period under blue LED light exposure, with a peak at 72 h, indicating an 8% increase of cells in G1 phase compared to the baseline at 0 h. Notably, the observation coincided with elevated cell-specific productivities under a light dose of 4 W*h/m^2^ (Fig. 3). This correlation aligns with independent studies showing that CHO cells in G1 phase exhibit enhanced productivity (Dutton et al. 2006; Park et al. 2016; Boer et al. 2004). Furthermore, the findings are consistent with other strategies to increase cell-specific productivity by arresting cells in G1 phase. In these approaches, cultivation conditions were shifted on purpose to install hypothermia (Becerra et al. 2012; Sunley et al. 2008; Fox et al. 2005; Kaufmann et al. 1999), hyperosmolality (Alhuthali et al. 2021; Min Soo Kim et al. 2002), or by adding chemical inducers (Park et al. 2016).

The mechanisms how blue light impacts cellular metabolism and the cell cycle remain speculative. Our data disclose cell arrest in the G1 phase and increased ROS levels. But both are unlikely to serve as single key impact factors. It is known that blue light generates intracellular superoxides because of the sensitivity of flavins to this wavelength (Nakashima et al. 2017). In turn, ROS can activate various signaling pathways, such as the NF-κB that link to cell proliferation and the cell cycle (Morgan and Liu 2011; Ledoux and Perkins 2014). However, elevated ROS levels alone are unlikely to account for the raised csp, as our data show even higher intracellular ROS levels at lower light doses that do not correlate with increased csp (Figs. 2d and 3). Most studies on the effects of blue light focus on retinal or plant cells, or on circadian rhythm regulation, which raises questions about their transferability to CHO production cell lines.

### Effect of blue light on medium components

To exclude the possibility that changes in the medium were responsible for the observed effect on csp, QTOF analysis of medium samples was conducted (Supplementary Information). Significant changes in medium components (defined as > 1.1-fold reduction) were identified and cross-referenced with databases and analyzed vitamin standards (Table S1). Out of 50 significantly downregulated components, only two—riboflavin and pyridoxine—were clearly identified based on correlation with measured standards and cross-references in the human metabolome database.

Riboflavin was completely degraded over 96 h in samples exposed to blue light (Fig. S2). This degradation is disadvantageous for cells, as riboflavin is an essential coenzyme for several dehydrogenases and oxidoreductases. Additionally, riboflavin, in the presence of light, can generate superoxides. However, extracellular measurements did not indicate elevated ROS levels under blue light conditions (Fig. S1).

A correlation of riboflavin deficiency and the cell cycle has been described by Long et al. (2018). However, instead of arresting the cell in the cell cycle, Long et al. observed that riboflavin deficiency promoted cell cycle progression, shortening the cycle and enhancing colony formation for 10–20 generations (Long et al. 2018).

Furthermore, a slight reduction in pyridoxine was observed (Fig. S3). While initially no pyridoxal is present in the medium, there seems to be a conversion from pyridoxine to pyridoxal in samples that have been illuminated by blue light (Fig. S4). Pyridoxine and pyridoxal are both utilized by cells to form pyridoxal phosphate, a cofactor for many reactions, including decarboxylation, deamination, and transamination (Liang et al. 2019). However, pyridoxal is chemically less stable and can react with primary amines (e.g., amino acids) to form Schiff bases. Chakravaty et al. have shown that iron(III)-pyridoxal complexes exhibit high phototoxicity, suggesting that elevated pyridoxal concentrations may be more detrimental compared to pyridoxine (Basu et al. 2015).

As no other compounds could be identified, the QTOF analysis results indicate that the increase in csp is likely due to the direct impact of blue light on the cells rather than indirect effects mediated by changes in the medium.

## Outlook

To translate early-stage findings into bioreactor applications, experimental conditions should be comparable, particularly concerning the optimum of light exposure identified in this study. The results observed in suspension cultures should be applicable to the screening conditions used in early-stage experiments. However, this translation may pose challenges, as researchers must account for differences in light exposure and growth kinetics between suspension cultures and adherent cells, as well as between high cell densities and uniform monolayers.

Surprisingly, our study observed a significant increase in cell-specific productivity in CHO cells when exposed to blue LED light at a specific dose. These findings have the potential to be leveraged for strain engineering of production cell lines to enhance productivity further. Additionally, incorporating an optogenetic switch could offer an opportunity to optimize this approach, combining genetic modifications with the intrinsic productivity increase induced by blue light.

Further research is required to determine the optimal conditions for implementing these findings within a bioprocess environment. Due to the observed growth reduction, application strategies either focus on fine tuning the light exposure or on explicitly optimizing product formation at low growth conditions in analogy to the application of mild hypothermia.

The surprising findings led to the German Patent Application #DE 10 2023 108 170.5.

## Supplementary Information

Below is the link to the electronic supplementary material.

Supplementary file1 (PDF 305 KB)

## Data Availability

The authors declare that the data supporting the findings of this study are available https://doi.org/10.18419/darus-4449 via the data repository of the University of Stuttgart. Raw data of the QTOF analysis were not uploaded due to size. However, files will be provided by the corresponding author upon reasonable request.
