# Supplementary material for: Optimum blue light exposure: a means to increase cell-specific productivity in Chinese hamster ovary cells
Source: Appl Microbiol Biotechnol. 2024 Dec 5;108:530. doi: 10.1007/s00253-024-13363-4 (PMC11621146; doi:10.1007/s00253-024-13363-4)
Supplement: Supplementary file 1 [file 253_2024_13363_MOESM1_ESM.pdf]

Optimum Blue Light Exposure: A Means to Increase Cell-Specific Productivity in Chinese Hamster Ovary Cells

Applied Microbiology and Biotechnology Journal

Keywords: CHO cells, cell culture, recombinant proteins, antibodies, cell-specific productivity

Stefanie Föller[1,*], Niklas Hetterscheidt[1], Levin Latastor[2], Gerald Radziwill[2], Ralf Takors[1,*]

[1]Institute of Biochemical Engineering, University of Stuttgart, Stuttgart, Germany

[2]Institute of Biology II, University of Freiburg, Freiburg, Germany

*corresponding authors:

Prof. Ralf Takors, takors@ibvt.uni-stuttgart.de

Stefanie Föller stefanie.foeller@ibvt.uni-stuttgart.de

Address: Allmandring 31, 70569 Stuttgart, Germany

Supplementary Information

Supplementary Methods

Detection and relative quantification of extracellular ROS

CHO DP-12 ells were cultivated in 50 mL mini bioreactors (Corning) and illuminated with 4 W*h/m² blue light (12 W/m² 10 min) or kept in the dark (dark control), respectively. After 96 hours an endpoint measurement of extracellular ROS with the OxyBURST™ Green H2HFF reagent (Invitrogen). Samples were centrifuged and supernatant was pipetted onto a black 96 well plate. 10 µg/mL OxyBurst were added to the supernatant samples, incubated for 2 minutes at 37°C and fluorescence measured at Ex:488nm/Em:530nm (Tecan). The fluorescence signal measured in the dark control served as the baseline.

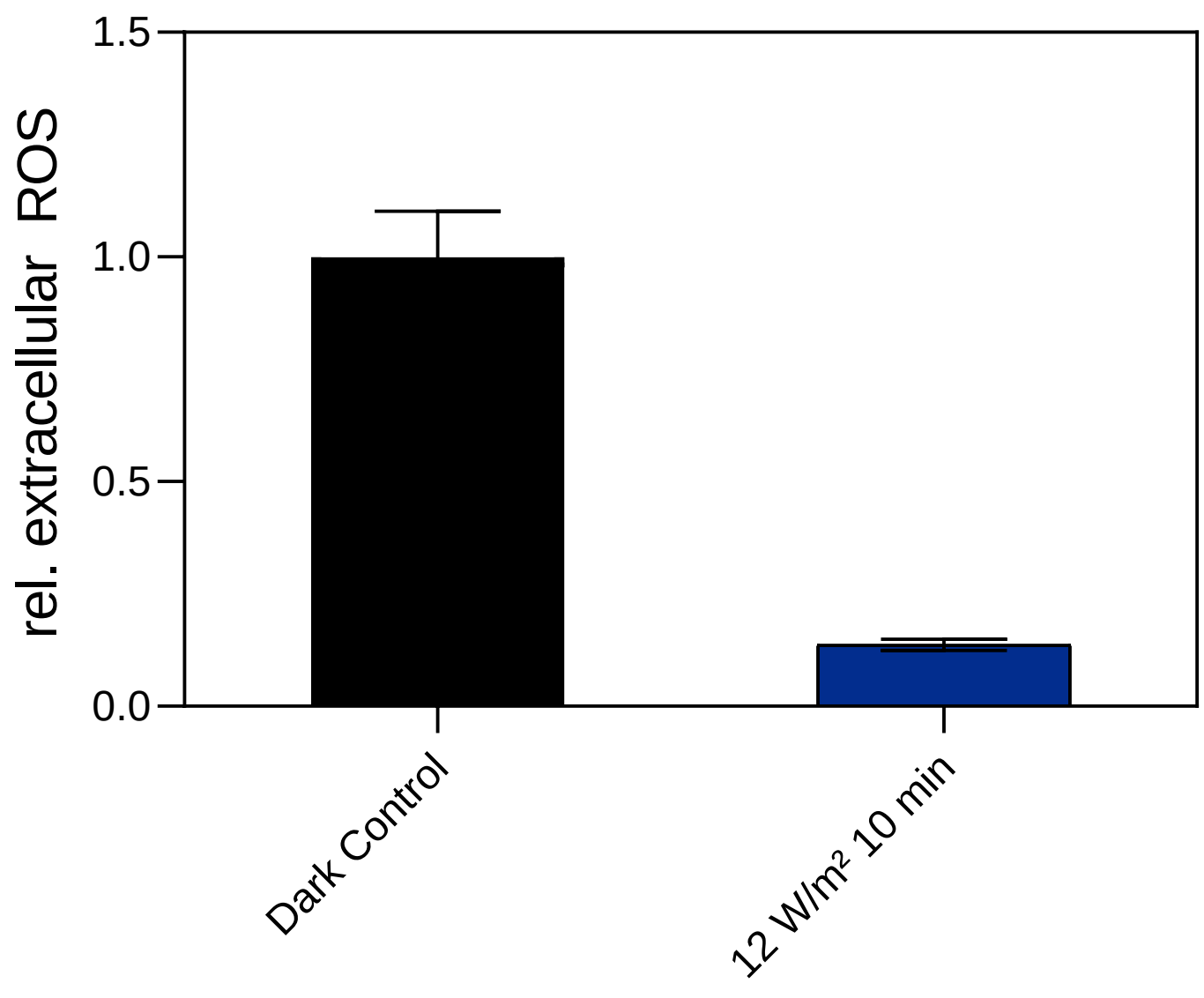

Fig. S1 Relative extracellular ROS levels for cells illuminated with 4 W*h/m² blue light compared to dark controls. n=3

LC-QTOF-MS Analysis for qualitative assessment of medium components

To ensure medium integrity despite light exposure, qualitative and untargeted LC-QTOF-MS measurements were conducted. These were performed on an Agilent 6540B Q-TOF Mass spectrometer in MS mode adjusted to extended dynamic range and low mass range (Feith et al., 2019). Only parent Ions were observed (CE = 0) at a constant fragmentor value. For ionization, an Agilent JetStream ESI source in positive polarity was used (Feith et al., 2019). Samples were separated chromatographically via HILIC (Feith et al., 2019; Teleki et al., 2015). The method was reduced to 40 minutes by steepening the gradient over just 20 minutes and reducing post analysis equilibrium time. Samples and standards were also constituted according to the procedure described in literature.

Since most common cell culture medium components, such as amino acids and carbohydrates are not described to be particularly susceptible to photon damage, special emphasis was placed on the investigation of water-soluble vitamins. Therefore, in addition to an untargeted comparison of mass spectra derived from light exposed and non-light exposed samples, standard samples of 8 vitamins (Table S1) were measured to facilitate identification of potential targets. Target identification for standard analytes was conducted two-factorially by checking nominal parent ion mass for [M+H]+ ions as well as retention time alignment (Δ_t^{max} = 0.2 min) using standard sample runs.

Beyond that, statistical analysis was performed on untargeted data to check for features deviating between conditions, beyond the limits of a predefined standard set. For this, significance testing via two-way-ANOVA (n=3, α= 0.05) and as fold change analysis (cutoff -1.1) between light exposed samples from the last and dark controls from the first day, was conducted. Resulted features were tested against different LC-MS databases (Massbank, HMDB) to check for further targets of light induced changes in the medium. No additional target was identified, leading to the conclusion, that apart from the discussed features, all medium components remained unaffected by light treatment, validating the deductions made from previously provided data.

Table S1 Water soluble vitamins used for standard runs with their exact mass and the nominal mass of their [M+H]+ ion used for analyte identification in untargeted media samples via retention time alignment.

**Standard Analyte**	**Mass [g/mol]**	**[M+H]+ (nominal) [m/z]**	**Retention time [min]**
Biotin	244.31	245	11.57
Pantothenic acid	219.24	220	11.69
Folic acid	441.40	442	N/A
p-Aminobenzoic acid	137.14	138	N/A
Pyridoxal	167.16	168	3.44
Pyridoxine	169.18	170	3.50
Riboflavin	376.37	377	5.58
Ascorbic acid	176.12	177	N/A

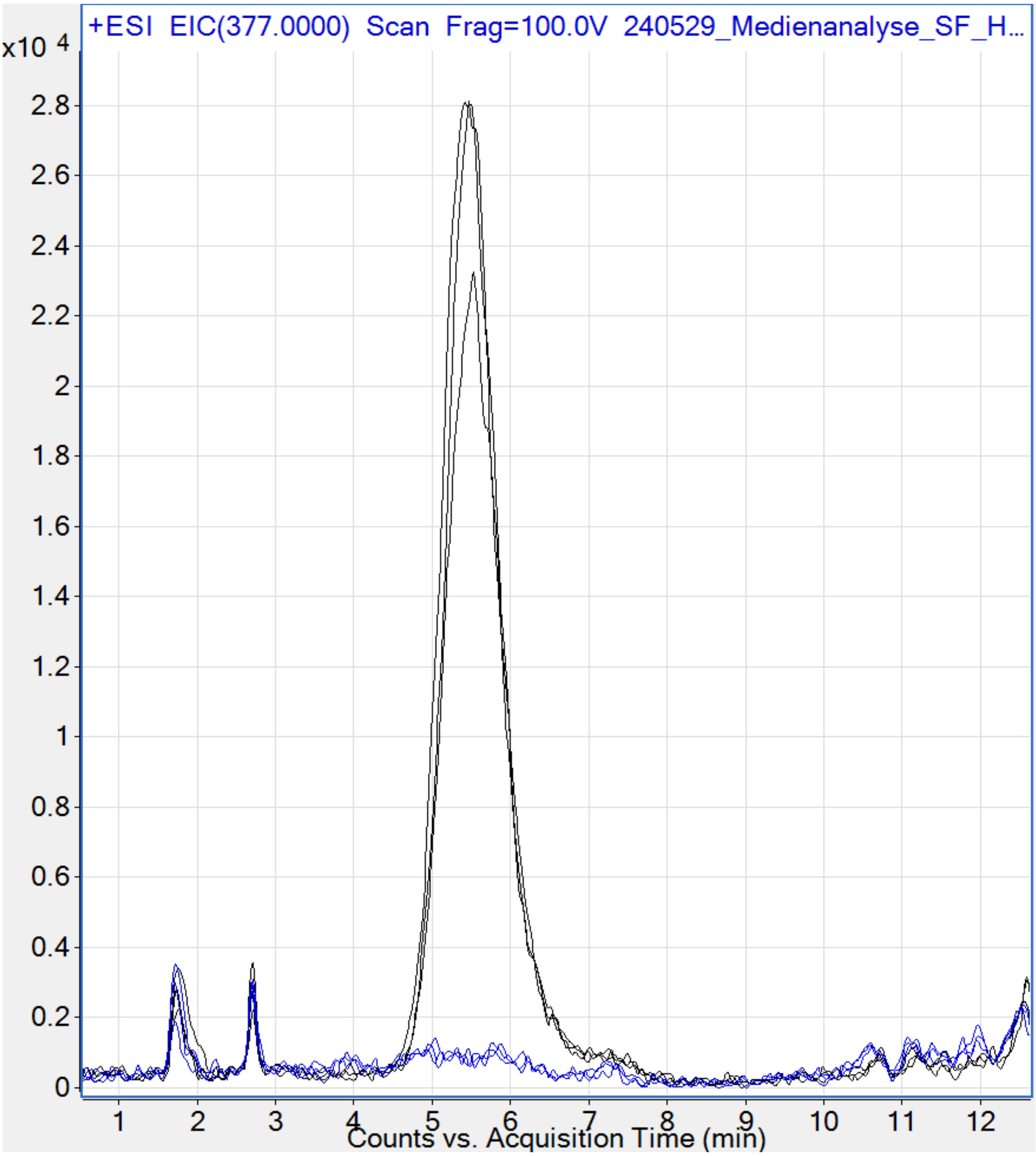

Fig. S2 Chromatograms of 377 000 m/z corresponding to a riboflavin standard at a retention time of 5.5 min. Shown are the chromatograms of samples that were kept in the dark (black and that were illuminated for 96 h with a light dose of 4 W*h/m² (blue). n=3

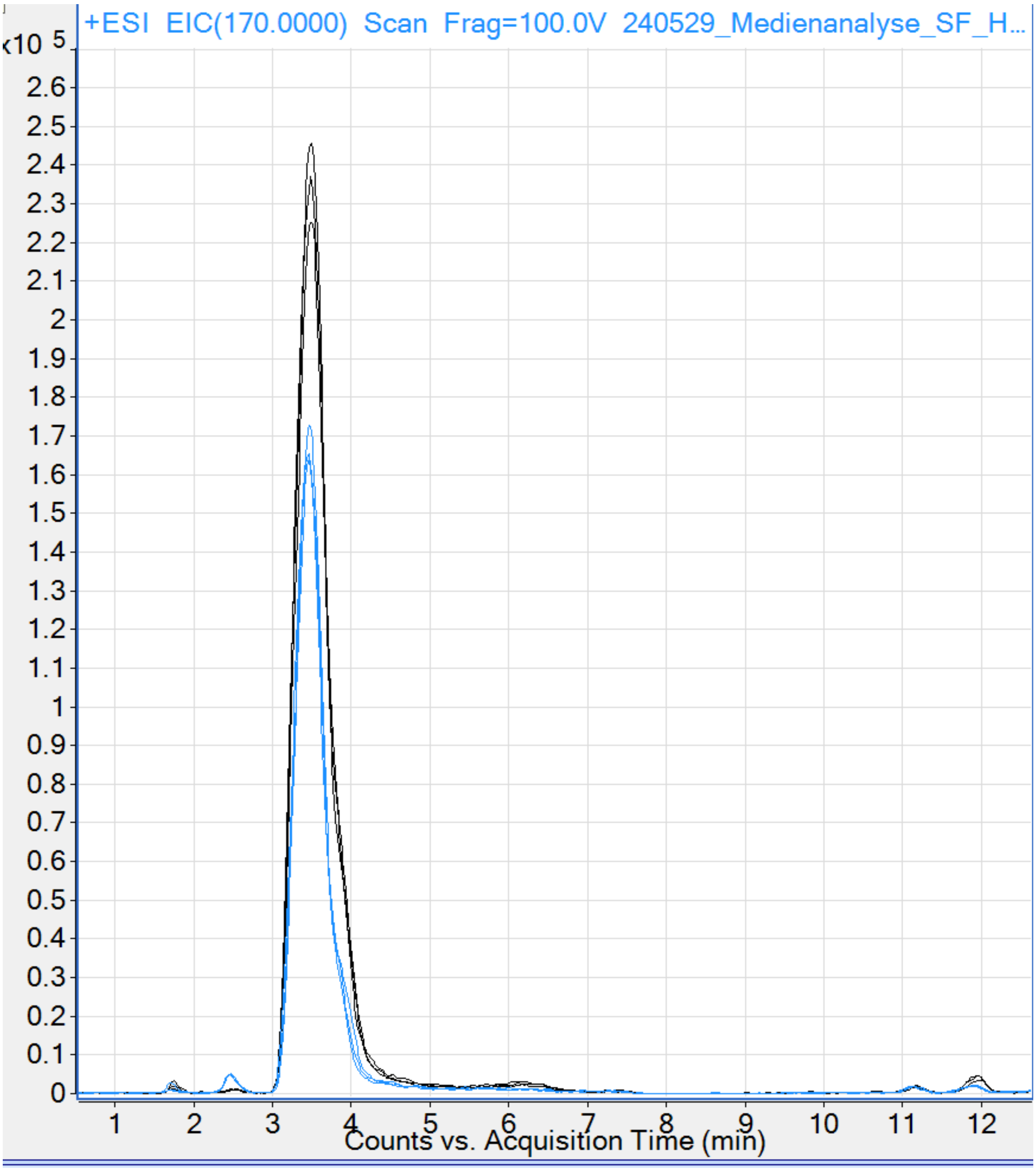

Fig. S3 Chromatograms of 170 000 m/z corresponding to a pyridoxine standard at a retention time of 3.5 min. Shown are the chromatograms of samples that were kept in the dark (black lines) and that were illuminated for 96 h with a light dose of 4 W*h/m² (blue). n=3

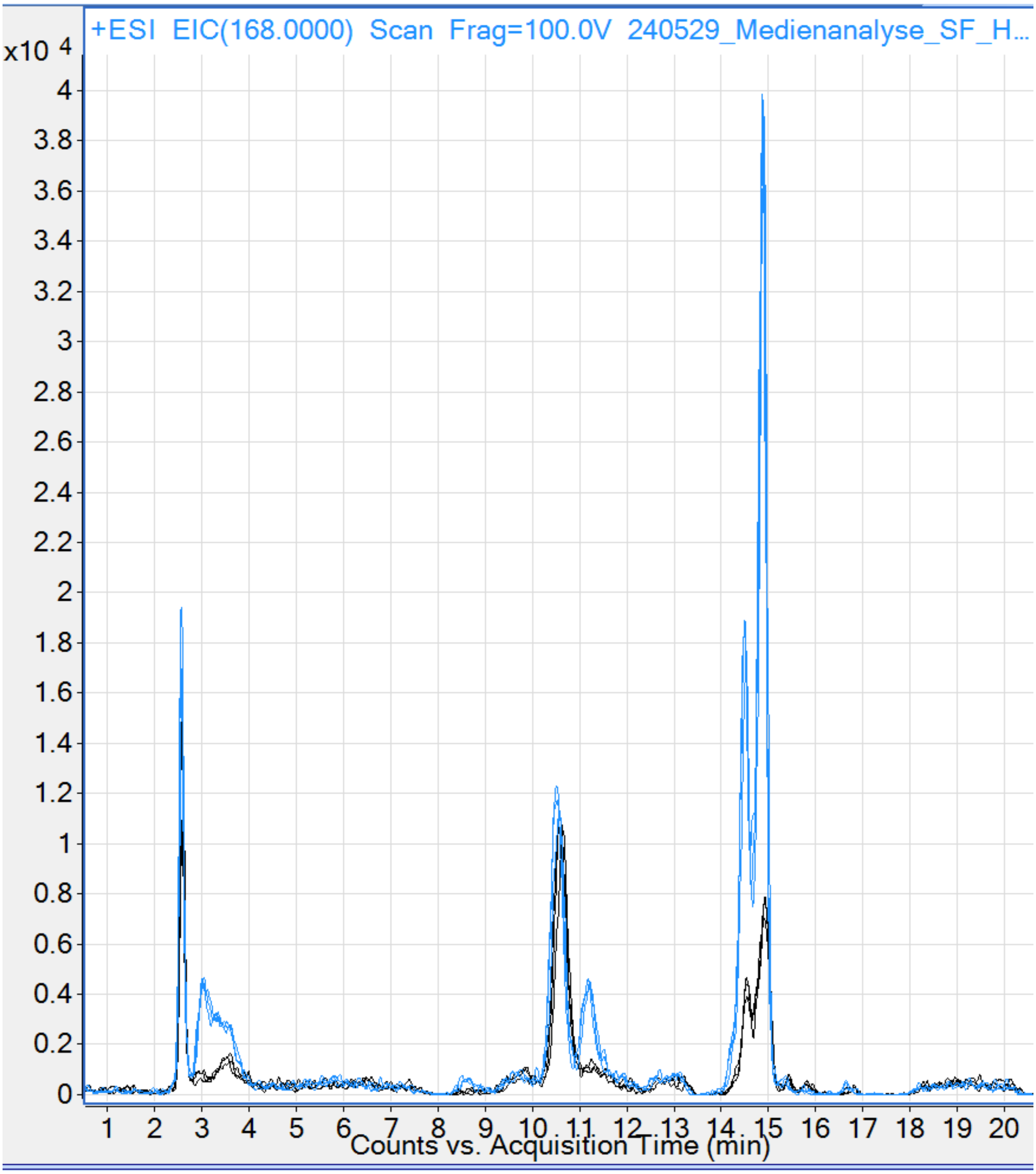

Fig. S4 Chromatograms of 168 000 m/z corresponding to a pyridoxal standard at a retention time of 3.5 min. Shown are the chromatograms of samples that were kept in the dark (black) and that were illuminated for 96 h with a light dose of 4 W*h/m² (blue). n=3

References

Feith, A.; Teleki, A.; Graf, M.; Favilli, L.; Takors, R. HILIC-Enabled 13C Metabolomics Strategies: Comparing Quantitative Precision and Spectral Accuracy of QTOF High- and QQQ Low-Resolution Mass Spectrometry. Metabolites 2019, 9, 63. https://doi.org/10.3390/metabo9040063

Teleki A, Sánchez-Kopper A, Takors R. Alkaline conditions in hydrophilic interaction liquid chromatography for intracellular metabolite quantification using tandem mass spectrometry. Anal Biochem. 2015 Apr 15;475:4-13. doi: 10.1016/j.ab.2015.01.002. Epub 2015 Jan 16. DOI: 10.1016/j.ab.2015.01.002
